# Genome engineering using a synthetic gene circuit in *Bacillus subtilis*

**DOI:** 10.1093/nar/gku1380

**Published:** 2014-12-30

**Authors:** Da-Eun Jeong, Seung-Hwan Park, Jae-Gu Pan, Eui-Joong Kim, Soo-Keun Choi

**Affiliations:** 1 Super-Bacteria Research Center, KRIBB, 125 Gwahak-ro, Yuseong-gu, Daejeon 305-806, Republic of Korea; 2 Biosystems and Bioengineering Program, University of Science and Technology (UST), 217 Gajung-ro, Yuseong-gu, Daejeon 305-350, Republic of Korea; 3 Genofocus Inc., 533-1 Yongsan-dong, Yuseong-gu, Daejeon 305-500, Republic of Korea

## Abstract

Genome engineering without leaving foreign DNA behind requires an efficient counter-selectable marker system. Here, we developed a genome engineering method in *Bacillus subtilis* using a synthetic gene circuit as a counter-selectable marker system. The system contained two repressible promoters (*B. subtilis xylA* (P_xyl_) and spac (P_spac_)) and two repressor genes (*lacI* and *xylR*). P_xyl_-*lacI* was integrated into the *B. subtilis* genome with a target gene containing a desired mutation. The *xylR* and P_spac_–chloramphenicol resistant genes (*cat*) were located on a helper plasmid. In the presence of xylose, repression of XylR by xylose induced LacI expression, the LacIs repressed the P_spac_ promoter and the cells become chloramphenicol sensitive. Thus, to survive in the presence of chloramphenicol, the cell must delete P_xyl_-*lacI* by recombination between the wild-type and mutated target genes. The recombination leads to mutation of the target gene. The remaining helper plasmid was removed easily under the chloramphenicol absent condition. In this study, we showed base insertion, deletion and point mutation of the *B. subtilis* genome without leaving any foreign DNA behind. Additionally, we successfully deleted a 2-kb gene (*amyE*) and a 38-kb operon (*ppsABCDE*). This method will be useful to construct designer *Bacillus* strains for various industrial applications.

## INTRODUCTION

*Bacillus* species are spore-forming Gram-positive bacteria and are the most frequently used bacteria for industrial enzyme production. Currently, about 60% of commercially available enzymes are produced from *Bacillus* species. *Bacillus* strains have also been used to produce nucleotides, vitamins, ribose and poly-γ-glutamic acid and as expression hosts to produce foreign recombination proteins (1–3). Industrial-scale production of commercial enzymes or metabolites requires strain engineering because wild-type *Bacillus* strains have not been adapted for overproducing specific enzymes or metabolites. Strain engineering often requires multiple mutations in the genome. However, the number of antibiotic selection markers available for use in *Bacillus subtilis* is limited. Thus, an effective method for genome engineering that is free of any antibiotic resistance markers is needed. Furthermore, the method must be usable to construct food-grade recombinant strains.

Genome modifications in *B. subtilis* have been achieved by using various positive selection markers, usually antibiotic-resistance markers. Traditional methods for the genome engineering without inserting any foreign DNA used negative selections that screened antibiotic-sensitive strains. Sometimes the mutant generation was based on the activity of a thermo-sensitive replication origin (4). However, the negative selections are laborious and time-consuming. Thus, a positive selection system using an efficient counter-selectable marker is required to facilitate genome engineering (5). Several counter-selectable markers based on the *upp* (6), *blaI* (7), *mazF* (8,9), *araR* (10) and *hewI* (11) genes have been used in *Bacillus* species. However, the method using *upp, blaI* or *araR* requires a strain with a specific mutation or insertion of a foreign gene in the chromosome. When these methods are applied to different strains, prerequisite mutants must be prepared. In the case of using toxic genes, such as *mazF* and *hewI*, tightly controlled expression is required. However, this is often unsuccessful and spontaneous resistant strains without the desired recombination are frequently generated. Thus, we developed a new and highly efficient marker-free method for *Bacillus* genome engineering without prior modifications of the bacterial strain.

Synthetic genetic circuits designed to program new biological behavior, dynamics and logic control have become valuable and widely applicable tools for studying genetics and cell biology. In addition to academic research, they have been applied commercially for the production of pharmaceuticals, biofuels and chemicals (12). In this study, we constructed a synthetic genetic circuit involving negative feedback loops; we also show its utility as a counter-selectable marker system. The system was demonstrated to be highly efficient for genome engineering in *B. subtilis* by constructing mutants having point modifications and deletions of a gene or an operon in the genome. Furthermore, the system showed that false-positive clones were generated infrequently.

## MATERIALS AND METHODS

### Strains and culture condition

The *B. subtilis* strains used in this study are listed in Table 1. *Escherichia coli* MC1061 was used to construct the recombinant plasmids. *Bacillus* cells were cultured in Luria-Bertani (LB), tryptic soy broth (TSB, Difco, Detroit, MI, USA) or trytic soy agar (TSA, Difco). To test the tryptophan auxotrophic phenotype, CHP minimal medium (13) supplemented with 1% glucose (CHPG1) was used. The cells were cultured in a 2× SG medium (14) for the protease assay. Transformation of *B. subtilis* was carried out by a method described previously (15). When required, the medium was supplemented with chloramphenicol (5 or 50 μg/ml), neomycin (10 μg/ml) or ampicillin (100 μg/ml).

**Table 1.** *Bacillus* strains used

Strain	Description	Source
168	trpC2	Laboratory stock
BS5417	BS168 thrC::P_xyl_-comK	This study
BS5465	BS168 + pA-xylR2	This study
BS5438	BS168 + pA-xylR2 + pUlac-trpC1028 (single crossover)	This study
BS5444	BS168 trpC^+^	This study
BS5446	BS168 trpC^+^ + pA-xylR2	This study
BS5449	BS168 trpC^+^ + pA-xylR2 + pUlac-aprE (single crossover)	This study
BS5450	BS168 trpC^+^ + pA-xylR2 + pUlac-nprE (single crossover)	This study
BS5451	BS168 trpC^+^ aprE^−^ + pA-xylR2	This study
BS5452	BS168 trpC^+^ nprE^−^ + pA-xylR2	This study
BS5456	BS168 trpC^+^ aprE^−^ + pA-xylR2 + pUlac-nprE (single crossover)	This study
BS5458	BS168 trpC^+^ aprE^−^ nprE^−^ + pA-xylR2	This study
BS5460	BS168 trpC^+^ aprE^−^	This study
BS5461	BS168 trpC^+^ nprE^−^	This study
BS5462	BS168 trpC^+^ aprE^−^ nprE^−^	This study
BS5589	BS168 + pA-xylR2 + pUlac-amyE (single crossover)	This study
BS5590	BS168 ΔamyE + pA-xylR2	This study
BS5591	BS168 + pA-xylR2 + pUlac-pps (single crossover)	This study
BS5592	BS168 Δpps + pA-xylR2	This study

### Plasmids

The primers used in this study are listed in Table 2. The helper plasmid (pA-xylR2) containing the *Bacillus* replication origin (rep), *xylR* repressor gene and spac promoter (P_spac_)–chloramphenicol resistant gene (*cat*) fusion cassette was constructed as follows. The rep fragment was obtained by a polymerase chain reaction (PCR) with primers rep-F1 and rep-R1 from plasmid pAD123 (16). The PCR product was digested with AatII and PvuII and inserted into the corresponding sites of plasmid pUC18 to construct pUC18-rep. *xylR* was amplified by PCR from plasmid pAX01 (17) with primers xylR-F1 and xylR-R3. Plasmid pUC18-rep was digested with BglII and NsiI, and the large fragment was fused to the *xylR* fragment using a cold fusion cloning kit (System Biosciences Inc., Mountain View, CA, USA) to construct pUC-*xylR*. The P_spac_ promoter was obtained by PCR from plasmid pMUTIN4 (18) with primers Pspac-F2 and Pspac-R1. The *cat* structural gene was amplified from the plasmid pAD123 with primers Cm-F3 and Cm-R3, and fused to the P_spac_ promoter by fusion PCR to obtain the P_spac_–*cat* cassette. The plasmid pUC-*xylR* was digested with SphI and NheI, and the large fragment was fused to the P_spac_–*cat* cassette using a cold fusion cloning kit to construct pA-*xylR*. The pA-*xylR* was digested with BglII followed by self-ligation and introduced into *B. subtilis* SCK6 (19) to obtain pA-*xylR2*. The integration vector (pUlac-*neo*) containing the neomycin resistant gene (*neo*), the *B. subtilis xylA* promoter (P_xyl_)-*lacI* fusion cassette and multiple cloning sites was constructed as follows. The P_xyl_ was obtained by PCR from the plasmid pAX01 with primers Pxyl-F3 and Pxyl-R2. *lacI* was amplified by PCR with primers lacI-F2 and lacI-R2 from pMUTIN4, and fused to P_xyl_ by fusion PCR to obtain the P_xyl_-*lacI* cassette. Plasmid pUC18 was digested with AatII and PvuII, and the large fragment was fused to the P_xyl_-*lacI* cassette using a cold fusion cloning kit to construct pUCxyl-*lacI*. The *neo* gene was amplified by PCR with primers neo-F5 and neo-R5 from plasmid pMLK83 (20), digested with HindIII and BamHI, and inserted into the corresponding sites of plasmid pUCxyl-*lacI* to construct pUlac-*neo*. To construct an insertion mutation in *trpC* of *B. subtilis*, the *trpC* gene from the chromosome of *B. subtilis* KCTC1028 was obtained by PCR with primers trpC-F2 and trpC-R2. The PCR fragment was digested with XhoI and XbaI and inserted into corresponding sites of pUlac-*neo* to construct the integration vector pUlac-*trpC1028*. The integration vector (pUlac-*aprE*) for the deletion mutation in *aprE* of *B. subtilis* 168 was constructed as follows. N- and C-terminus of *aprE* were amplified by PCR with primer sets aprE-D1/aprE-D2 and aprE-D3/aprE-D4, respectively, and fused into two PCR products by fusion PCR. The resulting *aprE* fragment was digested with XhoI and XbaI and inserted into corresponding sites of the pUlac-*neo* to construct integration vector pUlac-*aprE*. The integration vector (pUlac-*nprE*) for the point mutation in *nprE* of *B. subtilis* 168 was constructed as follows. The N- and C-termini of *nprE* were amplified by PCR with primer sets nprE-PM1/nprE-PM2 and nprE-PM3/nprE-PM4, respectively, and fused to two PCR products by fusion PCR. The resulting *nprE* fragment was digested with XhoI and XbaI, and inserted into corresponding sites of the pUlac-*neo* to construct the integration vector pUlac-*nprE*. The integration vector (pUlac-*amyE*) for the deletion of *amyE* of *B. subtilis* 168 was constructed as follows. N- and C-terminus of *amyE* were amplified by PCR with primer sets amyE-F5/amyE-R5 and amyE-F6/amyE-R6, respectively, and fused into two PCR products by fusion PCR. The resulting PCR fragment was digested with XhoI and XbaI and inserted into corresponding sites of the pUlac-*neo* to construct integration vector pUlac-*amyE*. The integration vector (pUlac-*pps*) for the deletion of *pps* operon of *B. subtilis* 168 was constructed as follows. N- and C-terminus of *pps* operon were amplified by PCR with primer sets pps-F1/pps-R1 and pps-F2/pps-R2, respectively, and fused into two PCR products by fusion PCR. The resulting PCR fragment was digested with XhoI and XbaI and inserted into corresponding sites of the pUlac-*neo* to construct integration vector pUlac-*pps*. The plasmids pA-xylR2 and pUlac-*neo* are available for the academic community, and their sequences were submitted to GenBank with the accession no. KP216525 and KP216524, respectively.

**Table 2.** Primers used

Primer	Sequence (5′ → 3′)^1^
rep-F1	ATAGACGTCAGATCTCGTACGATGCATAAACTGCATCCCTTA
rep-R1	ATACAGCTGGCTAGCATTATAGCATGCTATCCCACTTTATCCAATTTTC
xylR-F1	GTTTATGCATCGTACGCCGCGGGGATCCATGTTTATTTCAATGTTTTT
xylR-R3	CGAAAAGTGCCACCTGACGTCAGATCTTGATTAATTAATTCAGAACGC
Pspac-F2	GATAAAGTGGGATAGCATGCTACACAGCCCAGTCCAGACTATTCG
Pspac-R1	AATTGTTATCCGCTCACA
Cm-F3	ATTGTGAGCGGATAACAATTAAAAAGGATTGATTCTA
Cm-R3	GCCGATTCATTAATGCAGCTGGCTAGCAGATCTGCGAATGGCGACTAACGGGG
MCS	CGAAAAGTGCCACCTGACGTCACTAGTCTCGAGGCTAGCCCCGGGTCTAGACATATGGCATGCCTGC AGCGTACGGTCGACGAATTC
Pxyl-F3	AGCGTACGGTCGACGAATTCCTAAAAAAAACATTGAAATA
Pxyl-R2	TTGTCATTTCCCCCTTTGAT
lacI-F2	ATCAAAGGGGGAAATGACAAATGAAACCAGTAACGTTATACGA
lacI-R2	GCCGATTCATTAATGCAGCTGGGATCCTAATATAAGCTTCGGGAGCTGCATGTGTCAGA
Neo-F5	TATAAGCTTTCGAGATCAGGGAATGAGTT
Neo-R5	AAAGGATCCAATAAATACGTAACCAACAT
trpC-F2	ATACTCGAGGCAGCAGTTCCGCTTTATCT
trpC-R2	ATATCTAGAGCCTGTGATTCCGCCGCAAG
aprE-D1	ATACTCGAGTCATTGACACAGAAGAAAAC
aprE-D2	TTTTAGCTTTTTCATCCAATGT
aprE-D3	ACATTGGATGAAAAAGCTAAAAGAATTGAAAAAAGAT
aprE-D4	ATATCTAGACGTTGATTAACCCTTTTCCA
nprE-PM1	ATACTCGAGCAATACATAATGACTGAATA
nprE-PM2	GTTTATGCAGCAGATTGATT
nprE-PM3	AATCAATCTGCTGCATAAACAGATAACAGCCAAAAAGTCT
nprE-PM4	ATATCTAGAGGTCACGGGCAGACTGAATG
amyE-F3	ATAGTCGACTCAAATAAGGAGTGTCAAGA
amyE-R4	ATAATGCATGATGGTTTCTTTCGGTAAGT
amyE-F5	CCTGACGTCACTAGTCTCGAGGCGTGAATGGGAAAAATAAG
amyE-R5	TTTCAGCACTCGCAGCCGCC
amyE-F6	GGCGGCTGCGAGTGCTGAAATGAGGGCAAGGCTAGACGGGAC
amyE-R6	CAGGCATGCCATATGTCTAGAGTGAAGGAACTGTTCTTTTT
pps-F1	CCTGACGTCACTAGTCTCGAGAGATGGGGAAAGTGAAAAAA
pps-R1	GAAAAAAGCAGAAAAATGAC
pps-F2	GTCATTTTTCTGCTTTTTTCTAAAGCGGATTAGCGGACAG
pps-R2	CAGGCATGCCATATGTCTAGAGGAATGCCTGGATGATAATA
pps-F3	CAAAAACCGGATCGCTCAGT
pps-R3	CGAAAAAAGTCCTAAAGCAT
ppsA-F1	TGTTTTAGATCCGCATTTAGC
ppsA-R1	TCGTTCCTGACGTATAAATG
ppsB-F1	GGCATTAAGCGTGGAGAGTG
ppsB-R1	GCCGTTACCCCTTTTACCAA
ppsE-F1	CACTAATGAATCCGTGAAGA
ppsE-R1	TTTCGTTAAGCCTGTATGCC

^1^Underlines indicate restriction enzyme sites.

### Protease assay

*Bacillus* cells cultured in 1 ml of LB medium at 37°C for 16 h were inoculated in 10 ml of a 2× SG medium. After further culturing for 7 h at 37°C, the cultures were centrifuged to obtain the supernatants. Next, azocasein (Sigma, St. Louis, MO, USA) was dissolved at a concentration of 2% in an assay buffer containing 0.1 M sodium chloride, 0.01 M PIPES, 1 × 10^−6^ M zinc acetate and 5 mM calcium chloride (pH 7). The azocasein solution (300 μl) was then mixed with 100 μl of the supernatants from the *Bacillus* cultures and incubated in a 37°C water bath for 1 h. The reactions were stopped by adding 1.2 ml of 10% trichloroacetic acid, after which the reaction mixtures were allowed to stand at ambient temperature for 5 min. The tubes were then centrifuged for 3 min at 8000 × *g*, after which 600 μl of each supernatant was added to 700 μl of 1 M NaOH. The absorbance at 440 nm was then determined using a spectrophotometer.

## RESULTS

### Construction of a synthetic gene circuit

**Figure 1.**
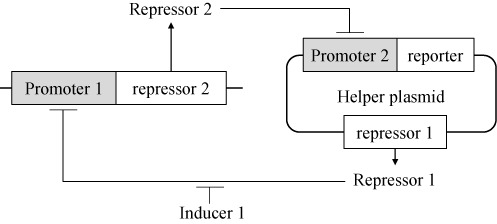
A synthetic gene circuit containing two repressible promoters and two repressor genes for a counter-selectable marker system during genome engineering of *B. subtilis*.

The regulatory network of synthetic gene circuits is artificial and can function as a molecular switch. The controllable switch can be applicable to signal a pop-in or pop-out of specific genes from chromosomes. In this study, a genetic circuit was constructed for the genome engineering of *B. subtilis* as a counter-selectable marker system. The system contained two repressible promoters and two repressor genes (Figure 1). Repressor 2 was transcribed from the promoter 1, and promoter 2 was located upstream of the reporter. Repressor 1 was expressed constitutively. In the absence of the inducer, the expressed repressor 1 repressed expression of repressor 2 from promoter 1, resulting in overexpression of the reporter. When inducer 1 was added to the system, promoter 1 was released from repression of the repressor 1 and overexpressed repressor 2. Repressor 2 blocked expression of the reporter from promoter 2. Thus, the promoter 1-repressor 2 cassette should be deleted to express the reporter, and could be used as a counter-selectable marker. The promoter 2-reporter cassette and repressor 1 gene were located on the plasmid so they could be removed later. We used *B. subtilis xylA* (P_xyl_) and spac (P_spac_) promoters for promoters 1 and 2, respectively. P_xyl_ can be induced by xylose. We also used *xylR, lacI*, and *cat* genes as the repressor 1, 2 and reporter genes, respectively.

### Construction of an insertion mutant

**Figure 2.**
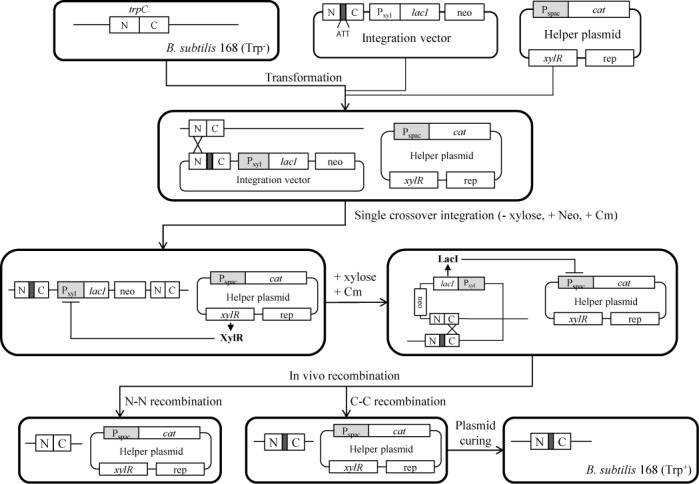
Strategy for constructing the *B. subtilis* 168 Trp^+^ strain using a synthetic gene circuit. The integration vector contains the *B. subtilis trpC* gene harboring three missing bases (ATT), the P_xyl_-*lacI* cassette and the neomycin-resistant gene (*neo*). N and C represent N- and C-terminal parts of the *B. subtilis trpC* gene. The helper plasmid contains a *xylR* repressor gene, replication origin (rep) for *Bacillus* and the P_spac_–*cat* fusion cassette. The integration vector was inserted into the *B. subtilis* 168 chromosome by single crossover integration. *In vivo* recombination between the N or C fragments under the presence of xylose and chloramphenicol (Cm) resulted in the deletion of the P_xyl_-*lacI* cassette and *neo*. The helper plasmid was removed to construct the *B. subtilis* Trp^+^ strain.

**Figure 3.**
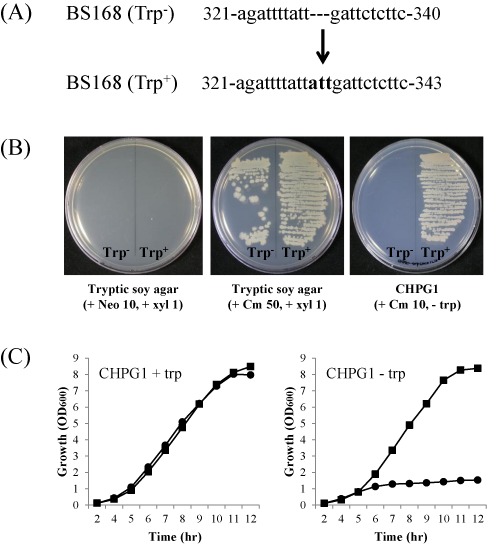
(A) Insertion of nucleotides into the *trpC* gene of *B. subtilis* 168 to construct the *B. subtilis* 168 Trp^+^ strain. The numbers indicate nucleic acid sequence positions relative to the first nucleotide of the start codon. (B) Growth of *B. subtilis* 168 Trp^+^ harboring the helper plasmid. Growth inhibition of the Trp^+^ strain on TSA containing neomycin indicated that the P_xyl_-*lacI* cassette and *neo* were deleted during *in vivo* recombination. Growth on TSA containing chloramphenicol revealed the presence of the helper plasmid. The Trp^+^ strain can be grown on a defined medium without a tryptophan supplement. (C) Growth of the Trp^−^ (black circles) and Trp^+^ (black squares) strains in a defined medium with or without a tryptophan supplement.

*B. subtilis* 168 requires tryptophan to grow in a minimal medium. Sequence analysis of *trp* operon revealed that the tryptophan requirement phenotype (Trp^−^) of the 168 strain came from a three base (ATT) deletion in the *trpC* gene (21). To obtain a Trp^+^ revertant, the bases (ATT) were inserted in the missing region of the *trpC* gene (Figure 3A). The scheme for constructing the Trp^+^ strain is depicted in Figure 2. The *trpC* gene harboring the missing three bases (ATT) was cloned into the integration vector containing the P_xyl_-*lacI* cassette and neomycin resistant gene (*neo*). The vector was inserted into the *B. subtilis* 168 chromosome by single crossover integration. Then, the strain was transformed with the helper plasmid pA-xylR2. The resulting strain BS5438 showed resistance to both neomycin and chloramphenicol, because the constitutively expressed XylR repressed the *lacI* expression from the P_xyl_ promoter, and the repression led to overexpression of the *cat* gene from the P_spac_ promoter. If xylose were added to the cells, it would inhibit XylR function, and LacI would be overexpressed; *cat* expression from the P_spac_ promoter would be repressed so that the cells would now be chloramphenicol sensitive. Thus, the Pxyl-*lacI* cassette should be deleted to grow the cells in the presence of chloramphenicol. The deletion occurred by recombination between N or C fragments in Figure 2. The *neo* gene was also deleted by the recombination. To demonstrate this hypothesis, strain BS5438 was cultured overnight in 1 ml of TSB containing a high concentration of chloramphenicol (50 μg/ml) and 1% xylose at 37°C. After spreading the cultured cells onto a TSA plate containing 50 μg/ml chloramphenicol and 1% of xylose, the cells were cultured overnight at 37°C. One hundred colonies were randomly selected and grown on TSA containing 10 mg/ml neomycin or 5 mg/ml chloramphenicol. Among them, 99 colonies displayed the neomycin-sensitive phenotype, indicating that xylose efficiently induced the recombination to delete the P_xyl_-*lacI* cassette and *neo* gene. Only 1% of the colonies were false positives. Recombination can occur with equal frequency between N or C fragments. One recombination returns the cells to the wild-type, and the other generates a mutation. When the randomly selected 100 colonies were cultured in a CHPG1 medium without tryptophan, 88 colonies were grown, indicating that 88% of the colonies were converted to the Trp^+^ phenotype. The *trpC2* genes from the four Trp^+^ strains were amplified by PCR with primers trpC-F2 and trpC-R2, and were analyzed by sequencing. The result showed that all of them contained the three base (ATT) insertion into the *trpC* gene, which restored TrpC function. The resulting strain BS5446 was neomycin-sensitive, chloramphenicol-resistant and the Trp^+^ phenotypes (Figure 3B), and could grow in a minimal medium without adding tryptophan (Figure 3C).

To remove the helper plasmid, the Trp^+^ strain BS5446 containing the helper plasmid pA-xylR2 was cultured in 1 ml of TSB for 24 h at 37°C, spread onto a TSA plate and further cultured overnight at 37°C. Then, 100 randomly selected colonies were picked and cultured on TSA or TSA containing 5 μg/ml chloramphenicol to select chloramphenicol-sensitive colonies. Among them, 36 colonies showed the chloramphenicol-sensitive phenotype, and no plasmids from them were detected. Thus, this result demonstrated that the helper plasmid could be easily removed.

### Construction of deletion and point mutants

**Figure 4.**
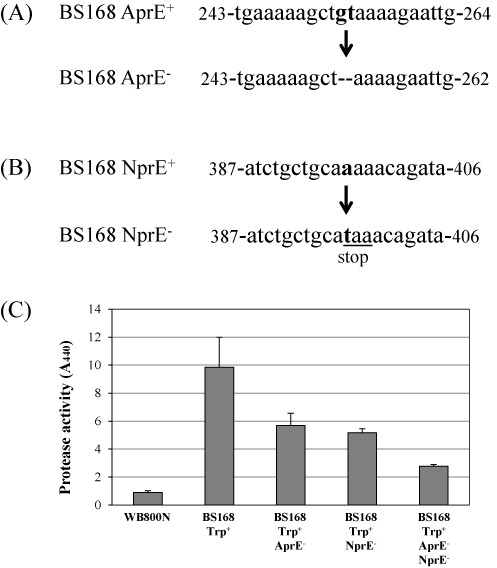
Deletion of nucleotides in the *aprE* gene (A) and point mutation in the *nprE* gene (B) of *Bacillus subtilis* 168 Trp^+^ strain to construct strains lacking AprE (AprE^−^) and NprE (NprE^−^). The numbers indicate nucleic acid sequence positions relative to the first nucleotide of the start codon. The point mutation created a stop codon in the NprE gene. (C) The protease assay of *B. subtilis* Trp^+^, AprE^−^ and NprE^−^ strains and a comparison of them with the WB800N strain in which eight extracellular proteases were deleted.

*B. subtilis* contains eight extracellular proteases (22). Among them, more than 95% of the extracellular proteolytic activity is contributed by the two major proteases, AprE and NprE (23). Inactivation of the two major proteases can significantly enhance the expression of heterologous proteins (24). We carried out the deletion and point mutations of *aprE* and *nprE*, respectively, by the method used in this study. For the deletion mutation, two bases (GT) were deleted from the *aprE* gene of *B. subtilis* 168 to construct the AprE^−^ strain (Figure 4A). To do this, the integration plasmid pUlac-*aprE* containing a two-base deletion in the *aprE* gene was introduced into strain BS5446 to integrate the plasmid into the chromosome. The resulting strain BS5449 was induced by xylose for recombination using the method described above. The recombinants showed neomycin-sensitive phenotypes in 42 clones among 50 randomly selected clones. The *aprE* genes were amplified by PCR from 10 randomly selected neomycin-sensitive clones. The nucleotide sequence analysis of the *aprE* genes showed that four clones contained deletion mutations in the *aprE* genes. The helper plasmid from the strain BS5451 (Trp^+^, AprE^−^) was removed by the method described above to construct strain BS5460.

For the point mutation, a single base in the *nprE* gene was changed to create a stop codon, which resulted in the NprE^−^ phenotype (Figure 4B). To do this, the integration plasmid pUlac-*nprE* was introduced into strain BS5446. The resulting strain BS5450 was induced by xylose for recombination using the method described above. The recombinants showed neomycin-sensitive phenotypes in 43 clones among 50 randomly selected clones. The *nprE* genes were amplified by PCR from nine randomly selected neomycin-sensitive clones. The nucleotide sequence analysis of the *nprE* genes showed that all nine clones contained point mutations in the *nprE* gene. The helper plasmid from strain BS5452 (Trp^+^, NprE^−^) was removed by the method described above to construct strain BS5461. Strain BS5462 (Trp^+^, AprE^−^, NprE^−^) was constructed by introducing the plasmid pUlac-*nprE* into strain BS5456, followed by inducing recombination and curing the helper plasmid. The deletion and point mutations in the *aprE* and *nprE* genes, respectively, were further confirmed by the protease assay in which extracellular protease activities were reduced in the mutants (Figure 4C).

### Deletion of a gene or operon

**Figure 5.**
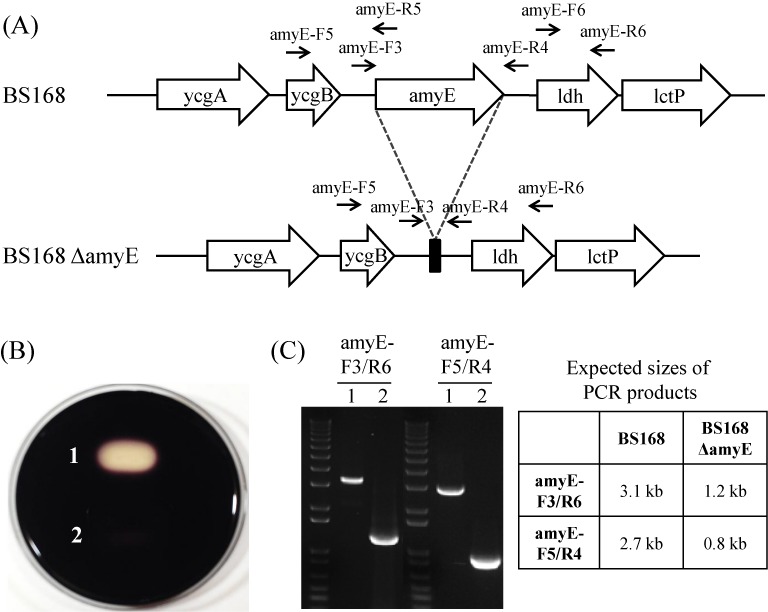
(A) Gene structures of wild-type (BS168) and deletion mutant of *amyE* gene (BS168 ΔamyE). Arrows above the genes indicate primer binding sites. (B) Amylase assay of BS168 (1) and BS ΔamyE (2) on an LB agar plate containing 1% starch. (C) PCR analyses of BS168 (1) and BS ΔamyE (2) with the indicated primer sets. Expected sizes of PCR products were shown in the table.

**Figure 6.**
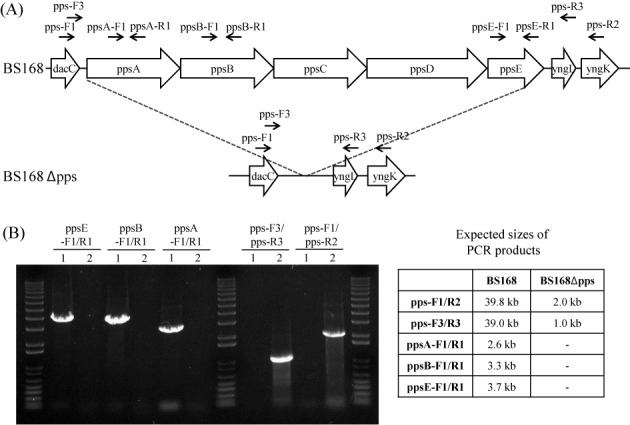
(A) Gene structures of wild-type (BS168) and deletion mutant of *pps* operon (BS168 Δpps). Arrows above the genes indicate primer binding sites. (B) PCR analyses of BS168 (1) and BS Δpps (2) with the indicated primer sets. Expected sizes of PCR products were shown in the table.

For the broad application of our method, we applied the method to the deletion of a gene and operon from the genome. To delete an about 2-kb *amyE* gene, the integration plasmid pUlac-*amyE* was introduced into strain BS5465. The resulting strain BS5589 was induced by xylose for recombination using the method described above. The recombinants showed neomycin-sensitive phenotypes in 25 clones among 25 randomly selected clones. Amylase assays with the selected clones on the LB agar plate containing 1% starch revealed that 16 clones (64%) showed amylase negative phenotypes. The deletion of the amylase gene was further confirmed by PCR (Figure 5). Next, we confirmed that large-sized DNA fragments can also be deleted by using our method. The deletion target was a 38-kb *pps* operon containing five large-sized genes (*ppsABCDE*). To delete the *pps* operon, the integration plasmid pUlac-*pps* was introduced into strain BS5465. The resulting strain BS5591 was induced by xylose for recombination using the method described above. The recombinants showed neomycin-sensitive phenotypes in 47 clones among 50 randomly selected clones. PCR analysis revealed that 3 of the 47 neomycin-sensitive clones (6.4%) contained a deleted *pps* operon (Figure 6). The results confirmed that our method is a powerful tool for *Bacillus* genome engineering.

## DISCUSSION

A synthetic gene circuit has been designed to construct logical devices including timers, counters, clocks, logic processors, pattern detectors and intercellular communication modules, and can be applied to various research, industrial and medical fields (25,26). Here, we designed a synthetic gene circuit for a counter-selectable marker system to engineer a genome. Some previously reported counter-selectable marker systems required prerequisite mutations or insertions of foreign genes into the chromosome, such as inactivation of the native *upp* gene, or replacement of the native *araR* gene with the Para-*neo* cassette (6,7,10). In a method that uses Xer recombination, the dif site, which is a recognition sequence for the native Xer site-specific recombinases, remained in the chromosome during deletion of the selectable marker gene (27). However, the remaining exogenous non-native sequences in the chromosome hamper the system to apply to food-grade genome engineering. In our system, the reporter system (P_spac_-*cat* cassette) was located on the plasmid. After engineering the target gene, the plasmid can be removed easily from the cell. Thus, the system does not retain any foreign DNA fragments in the chromosome, and this enables the system to be applied to food-grade genome engineering.

*mazF* has been used as a counter-selectable marker without any residual exogenous sequences in the chromosome (8,9). *mazF* encodes an endoribonuclease that specifically cleaves ACA sequences of mRNAs, and is highly toxic when expressed in cells (28). Genome engineering using the *mazF* system resulted in many false-positive clones in our experimental condition, and was not a proper method. Recently, an improved method using a mini-*mazF*-cassette was reported (29). They used direct repeat (DR) sequences for the *in vivo* recombination in the *amyE* deletion and *gfp* insertion experiments, which resulted in the DR remaining in the chromosome during deletion of the selectable marker system. The report used a PCR cassette without the DR in the deletion of a large fragment. However, in this case, another DNA fragment for the *in vivo* recombination was need, and seven DNA fragments should be fused by PCR. The report emphasized that the toxic gene *mazF* was under the control of a *B. subtilis xyl* promoter, and its expression was able of tight control. In their system, the promoter is controlled by a host-encoded repressor, XylR, which showed considerable sequence similarities in the *Bacillus* group, suggesting that the mini-*mazF*-cassette system can possibly work in other *Bacillus* species (30). However, tight regulation of the promoter by host XylR is not guaranteed because the consensus sequences of the XylR binding sites vary in different subgroups of genomes (30). Our system contains *B. subtilis xylR* in a helper plasmid to control P_xyl_ in an integrative plasmid. Thus, our system can possibly be directly used in any transformable wild-type strains. In addition, the tightly controlled P_xyl_ by the XylR shows that our system is highly efficient and infrequently generates false positives.

In this system, the sizes of the ‘N’ and ‘C’ fragments for the recombination ranged approximately from 500 to 1100 base pairs. In a mini-*mazF*-cassette system, the authors used 500-bp fragments for the recombination (30). Previous reports showed that ∼70 bp of homology is required for detectable homologous recombination in *B. subtilis*, and the recombination frequency showed a linear dependence on substrate length within the range of 77–165 bp (31). Thus, for the efficient recombination, the sizes of the ‘N’ and ‘C’ fragments could be recommended to be greater than 200 base pairs.

Our system can be used to construct insertion, deletion and point mutations in the chromosome without genome information and prerequisite genetic background. Additionally, the selectable marker system is located on the plasmid, which can be easily removed later. These characteristics suggest that the system can be applied to food-grade engineering of other *Bacillus* species or previously used industrial *Bacillus* strains. Many *Bacillus* strains are being used in agriculture as biocontrol agents or biofertilizers. Live *Bacillus* cells are spread in the environment for agricultural application. Thus, food-grade strain engineering is indispensable for improving the agricultural *Bacillus* strains. Our system would be useful to engineer *Bacillus* strains for agricultural applications.
